# Thoracic and Lumbar Spine Dissection for Pediatric Deformity

**DOI:** 10.1016/j.jposna.2025.100213

**Published:** 2025-05-28

**Authors:** Ravi R. Agrawal, Keith Bridwell, Munish Gupta, Blake K. Montgomery

**Affiliations:** Department of Orthopaedic Surgery, Washington University School of Medicine, St. Louis Children's Hospital, St. Louis, MO, USA

**Keywords:** Spine, Dissection, Thoracic, Scoliosis, Education, Video, Durotomy, Pneumothorax

## Abstract

The posterior approach to the thoracic and lumbar spine remains the most commonly used method for treating idiopathic scoliosis (IS). A detailed understanding of the relevant anatomy reduces iatrogenic complications, such as durotomy and pneumothorax, while an efficient surgical technique minimizes operative time and blood loss. Few video-based resources detailing step-by-step exposure of the posterior elements are available. Such videos would enhance trainee preparation prior to posterior spinal fusion (PSF) for IS. This technique article reviews the authors’ preferred surgical approach, focusing on the pearls and pitfalls of errant techniques. The intended audience includes orthopaedic surgery and neurosurgery trainees. Additionally, it provides a sample pre-test to evaluate trainee knowledge preoperatively (see Appendix).

**Key concepts:**

(1) Subperiosteal dissection after splitting the apophysis is essential to achieving hemostasis.
(2) Errant dissection of the thoracic spine can cause durotomy, pneumothorax, and neurologic injury.
(3) Supraspinous ligament violation near the UIV can increase the risk of junctional kyphosis.
(4) Preserving the UIV and LIV facet joints is essential to maintain adjacent segment joint health.
(5) Safe placement of all spinal instrumentation (hooks, screws, and sublaminar fixation) requires adequate spinal exposure.

## Technique Video

Video 1

Video 2

Video 3

## Introduction

The posterior approach to the thoracic and lumbar spine has been documented since the early 20th century [[1], [2], [3]]. Understanding this technique is essential for treating IS, as all posterior-based instrumentation constructs continue to gain popularity [4,5]. Proficiency with this approach reduces the risk of iatrogenic intraoperative complications, such as pneumothorax, durotomy, spinal cord injury (SCI), nerve root injury, adjacent segment disease (ASD), proximal junctional kyphosis (PJK), and hemorrhage [[6], [7], [8], [9], [10]]. Effective dissection and careful hemostasis decrease operative time and blood loss, thus lowering the risk of postoperative infection and the need for blood transfusion [11,12]. The objectives of this approach are to safely and swiftly expose the posterior spinal column for subsequent instrumentation and deformity correction.

PSF for IS typically involves one surgeon and an assistant or two surgeons working simultaneously to achieve the dissection goals [[13], [14], [15]]. The second surgeon may be a surgical trainee, such as a resident with limited technical experience or knowledge regarding this approach. Few video-based resources are available for surgical trainees to use in preparation for these surgeries [16,17]. Currently, no surgical technique video focuses primarily on thoracic and lumbar spine dissection in pediatric spinal deformity. This article and the accompanying technique video were created to comprehensively detail the steps of the author's preferred method for performing the posterior approach to the thoracic and lumbar spine. The pitfalls of erroneous techniques are highlighted. The intended educational audience includes orthopaedic surgery and neurosurgery trainees. Lastly, a novel corresponding test was created to assess trainee knowledge, aiming to help attending surgeons address trainee knowledge gaps preoperatively.

## Methods

### Review of anatomy

**Figure 1:**
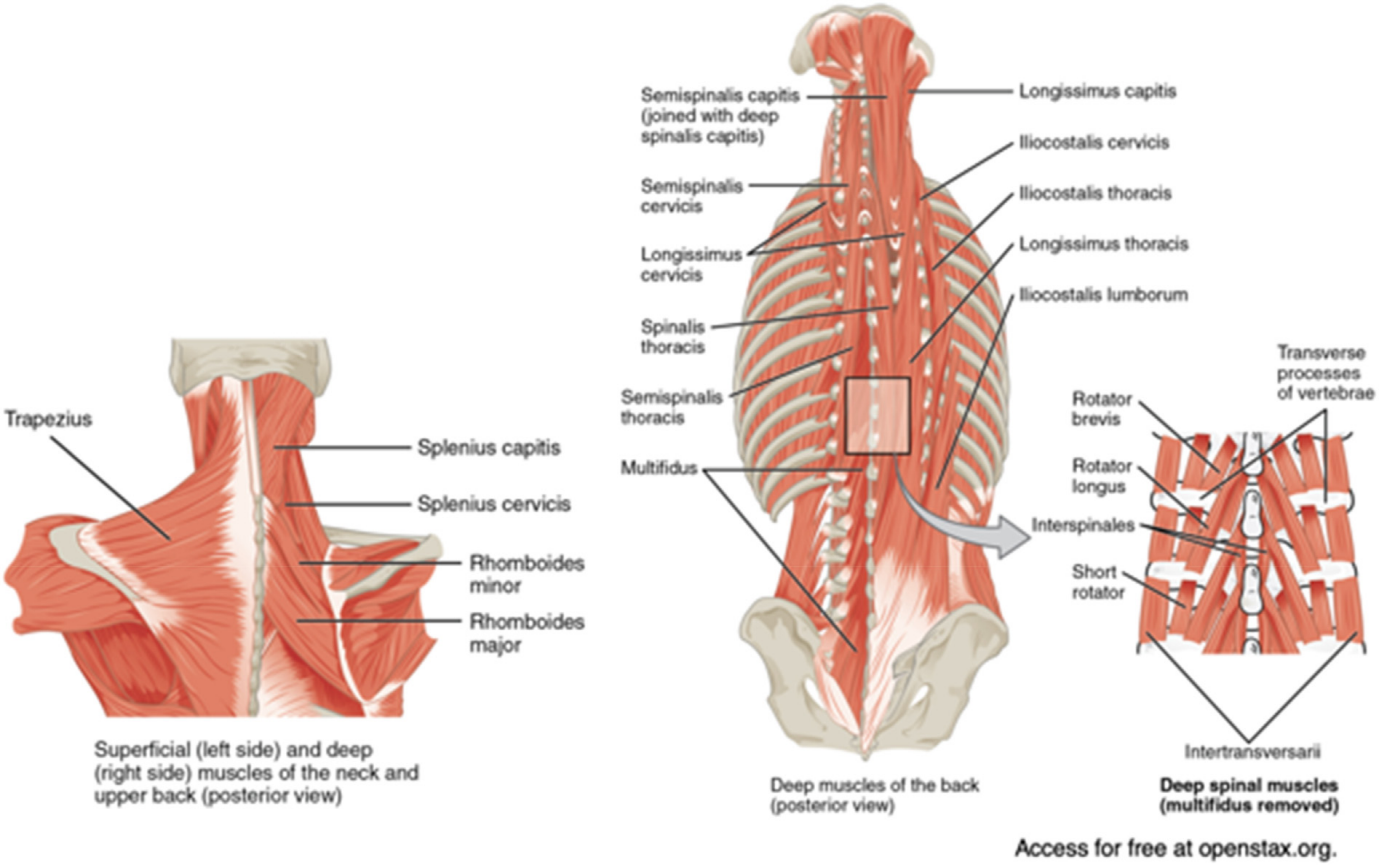
Demonstration of the three layers of dorsal paraspinal muscle. The superficial layer includes the trapezius, latissimus dorsi, and lumbodorsal fascia; the intermediate layer features the erector spinae and serratus posterior superior and inferior; and the deep layer encompasses the various muscle groups originating from the transverse processes (TPs) [24].

This approach is based on subperiosteal elevation of the paraspinal musculature as a single sleeve bilaterally off the posterior vertebral column. Three layers are described overlying the thoracic and lumbar vertebrae dorsally: the superficial, intermediate, and deep layers. The superficial layer consists of the trapezius, extending from the external occipital protuberance to the spinous processes of C7-T12, and the latissimus dorsi, originating from the spinous processes of T7-L5 and the iliac crest. The intermediate layer is composed of the erector spinae, which includes the spinalis, longissimus, and iliocostalis. The spinalis forms the medial border of the erector spinae, being elevated off the spinous processes. The serratus posterior superior and inferior arise from the cervicothoracic and thoracolumbar spinous processes in this layer as well. Lastly, the deep layer consists of the semispinalis thoracis, rotatores thoracis, multifidus thoracis, and intertransversarii. These muscles originate at the transverse processes (TPs) and spinous processes. They comprise the ‘intervening’ muscles between vertebral levels and must be elevated to visualize the TPs and lateral pars interarticularis (Fig. 1). The origins and insertions of the muscles encompassed by the three layers are demonstrated in Table 1 [27].

**Table 1.** Origins and insertions of relevant paraspinal musculature divided by layer.

Muscle	Origin	Insertion	Action
**Superficial**			
Trapezius	Occiput, nuchal ligament, C7-T12 spinous processes	Lateral third of the clavicle, acromion, scapular spine	Elevates, depresses, rotates scapula
Latissimus dorsi	T7-L5 spinous processes, thoracolumbar fascia, iliac crest, inferior angle of the scapula, and distal three ribs	Intertubercular sulcus of the humerus	Extends, adducts, internally rotates humerus
**Intermediate**			
Spinalis thoracis	T11-L2 spinous processes	T2-T8 spinous processes	Extension and lateral bending of vertebral column
Longissimus	Thoracis: T1-T12 TPs Lumborum: L1-L5 spinous processes and TPs, sacrum, iliac crest	Thoracis: C2–C6 TPs Lumborum: T1-T12 TPs and ribs	Extension and lateral bending of vertebral column
Iliocostalis	Thoracis: Angles of ribs 7-12 Lumborum: Iliac crest, sacrum	Thoracis: C7 TP and angles of ribs 1-6 Lumborum: L1-L4 TPs and angles of ribs 5-10	Extension and lateral bending of vertebral column
**Deep (Transversospinalis)**			
Semispinalis thoracis	T6-T10 TPs	C6-T4 spinous processes	Extension and rotation of vertebral column
Multifidus	Sacrum, PSIS, T1-T12 TPs, C4–C7 articular processes	Spinous process of cranial adjacent vertebrae	Extension, rotation, and stabilization of vertebral column
Rotatores spinae	Posterior and inferior aspects of thoracic and lumbar TPs	Lamina and TP of cranial adjacent vertebral level	Extension, rotation, and stabilization of vertebral column
Interspinales (thoracic and lumbar)	Thoracic and lumbar TPs	Superior aspect of cranial adjacent spinous process	Extension of vertebral column
Intertransversarii (thoracic and lumbar)	Thoracic and lumbar TPs	Inferior border of cranial adjacent TP	Lateral bending of the vertebral column

**Figure 2:**
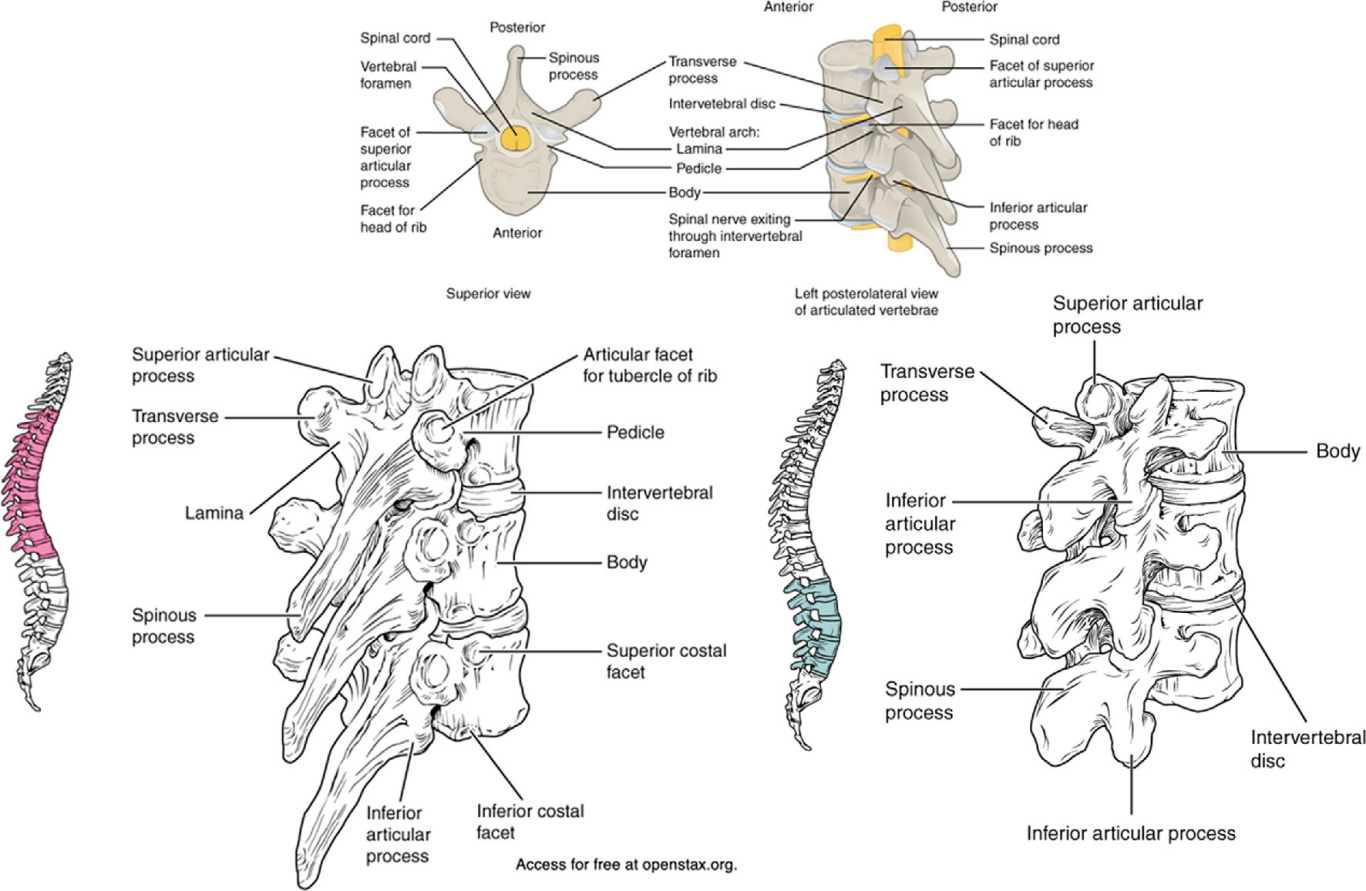
Demonstration of the osseous anatomy: Thoracic and lumbar vertebrae anatomy depicting the transverse process, lamina, pedicle, facet joints, and vertebral bodies. It also illustrates the global sagittal profile of the spine, showing the cranial-to-caudal change of the spinous processes [25].

Vertebral anatomy must be studied preoperatively for safe dissection (Fig. 2A and B). The spinous process, lamina, superior articular facet (SAP), inferior articular facet (IAP), and transverse process (TP) are key anatomic landmarks to identify. The morphology of each structure progressively varies from cranial to caudal along the thoracic and lumbar spine. Thoracic spinous processes are oriented caudally in the sagittal plane, while lumbar spinous processes are oriented more orthogonally to the longitudinal axis of the vertebral column. Additionally, the ribs connect to the thoracic vertebral bodies at the costal facets. The supraspinous ligament overlies all spinous processes and acts as the primary restraint against junctional kyphosis. The thick interspinous ligament connects each spinous process deep to the supraspinous ligament.

### Incision planning

After careful patient positioning to prevent compressive or traction neuropathy, along with the establishment of intravenous and arterial lines and the placement of neuromonitoring leads, incision planning may proceed. Fusion for IS can involve dissection from the upper thoracic spine to the lumbar spine, which necessitates knowledge of thoracic and lumbar surface landmarks. Thus, the large T1 spinous process is first palpated and marked, as it assists in the localization of the proximal level superficially. Distally, the iliac crests are marked. A tangential line connecting the dorsal apices of the iliac crests corresponds to the L4 vertebral level. Both caudal landmarks are helpful to identify on the trunk to aid in planning the caudal extent of the surgical incision. The ribs may be marked as a secondary check.

**Figure 3:**
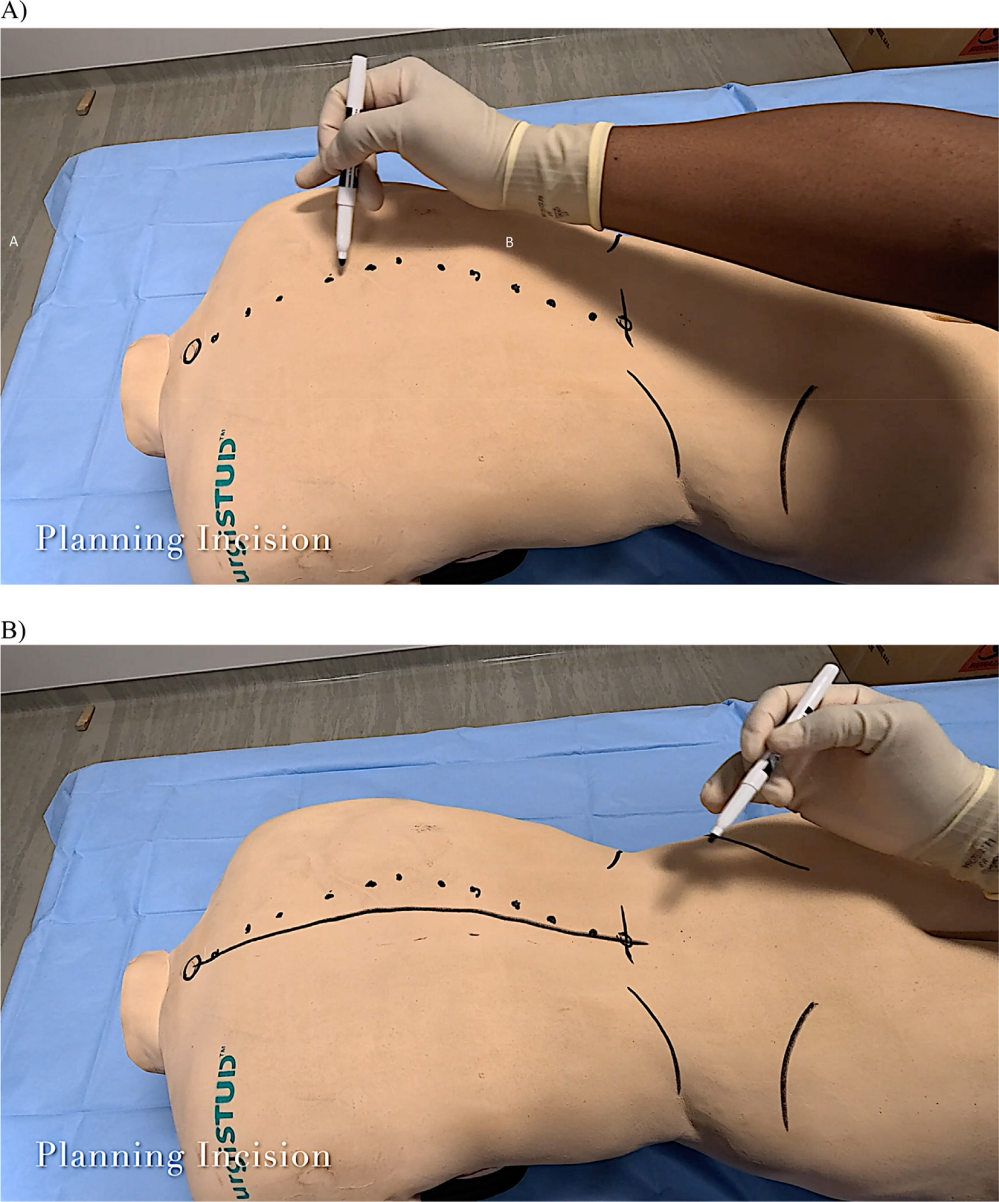
A) The large T1 spinous process is marked at the junction of the neck and shoulders. Marking the distal ribs and the iliac crests is optional. The spinous processes are then marked from cranial to caudal. B) A line to mark the intended incision is drawn midway between the apex of the spinous processes and a parallel longitudinal line running down the back.

The spinous processes from T1 to the lumbar spine are sequentially marked from cranial to caudal (Fig. 3A). The contour of these spinous processes should reflect the radiographic deformity observed. Depending on the patient's body habitus, the incision can be planned accordingly to ensure a straight scar postoperatively. Obese individuals will not have much surface correction, so a straight incision is preferred. It may be advisable to curve the incision in very slender individuals, where correction will alter the incision. In anticipation of spinal deformity correction, a longitudinal line is drawn midway between a line connecting the spinous processes and a straight line that runs longitudinally down the back (Fig. 3B).

### Superficial dissection

The primary surgeon typically stands on the left side of the prone patient, while the assistant is positioned on the right. This arrangement is due to the concavity of an idiopathic scoliosis curve, usually on the left side. Dissection and instrumentation around the concavity of this curve are considerably more precarious. The pedicles are narrower on the left side in the mid-thoracic spine, and the thecal sac is positioned closer to the medial pedicle walls [26].

**Figure 4:**
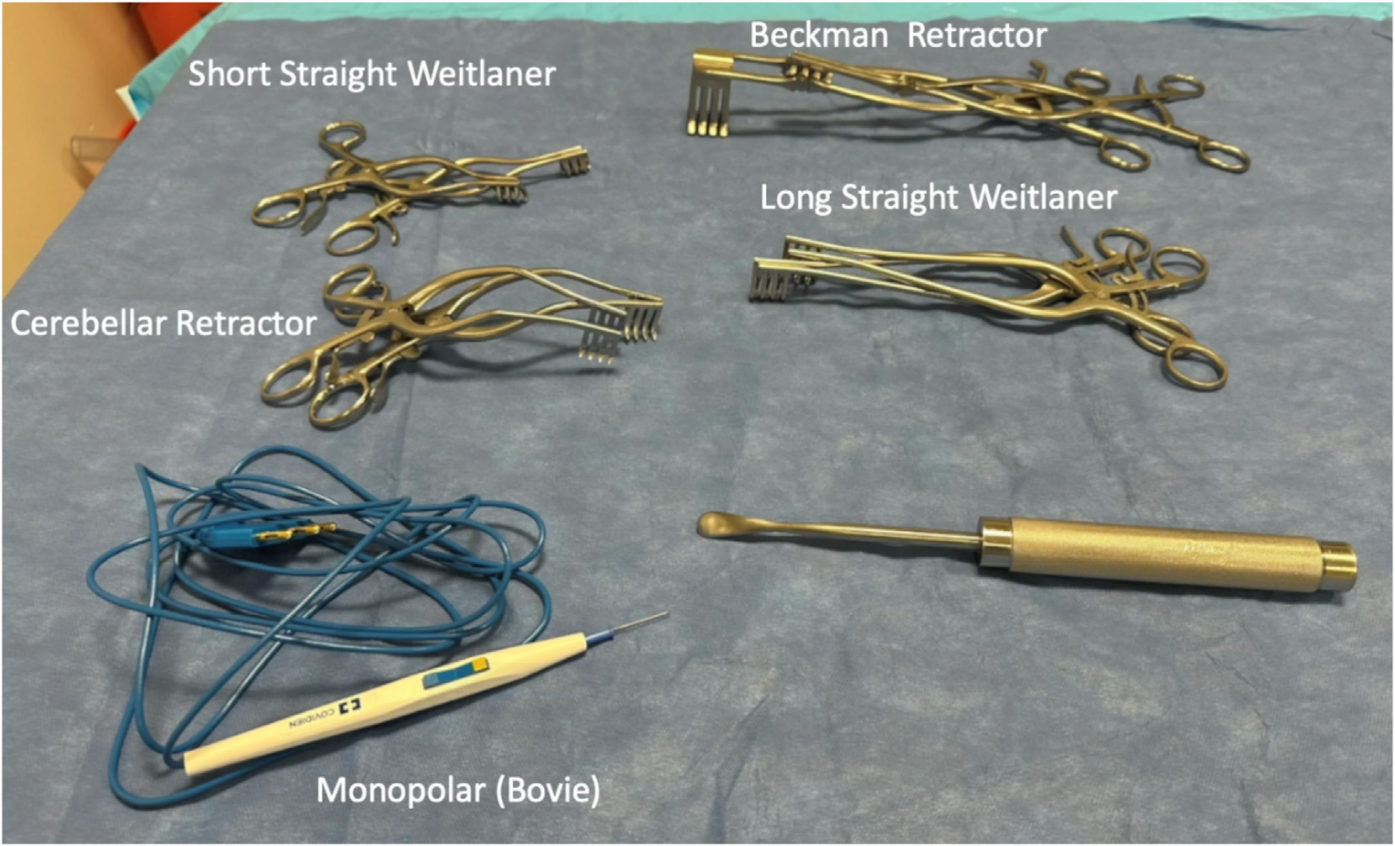
Essential instruments are assembled for thoracic spinal dissection.

The incision is confirmed, and hash marks are drawn orthogonal to the longitudinal incision to assist with eventual closure. Essential instruments to have readily available for spine dissection prior to incision include broad scalpel blades, short and long straight Weitlaner retractors, cerebellar retractors, Beckmann retractors, suction, monopolar electrocautery, Cobb elevators, sponges, and a variety of self-retaining retractors (Fig. 4). Bipolar cautery with saline coolant (e.g., Aquamantys Bipolar Sealers, Medtronic) may also be desirable for enhanced hemostatic control. A mixture of epinephrine and lidocaine can be injected around the planned incision to enhance hemostasis and reduce the requirement for hypotensive anesthetic intraoperatively.

The skin is then sharply divided with a broad blade, such as a #10 blade. The dissection is carried partially through the dermis. Cautery is then utilized for the remainder of the exposure. The dermis and superficial underlying fat are incised decisively in one contiguous layer to prevent disruption of perforating arteries supplying the dorsal skin. Creating a single plane of tissue dissection from the skin to the lumbodorsal fascia and spinous processes aids in successful wound closure of thick tissue flaps. This mitigates the risk of wound dehiscence and seroma formation that occurs from the iatrogenic creation of multiple soft tissue planes.

**Figure 5:**
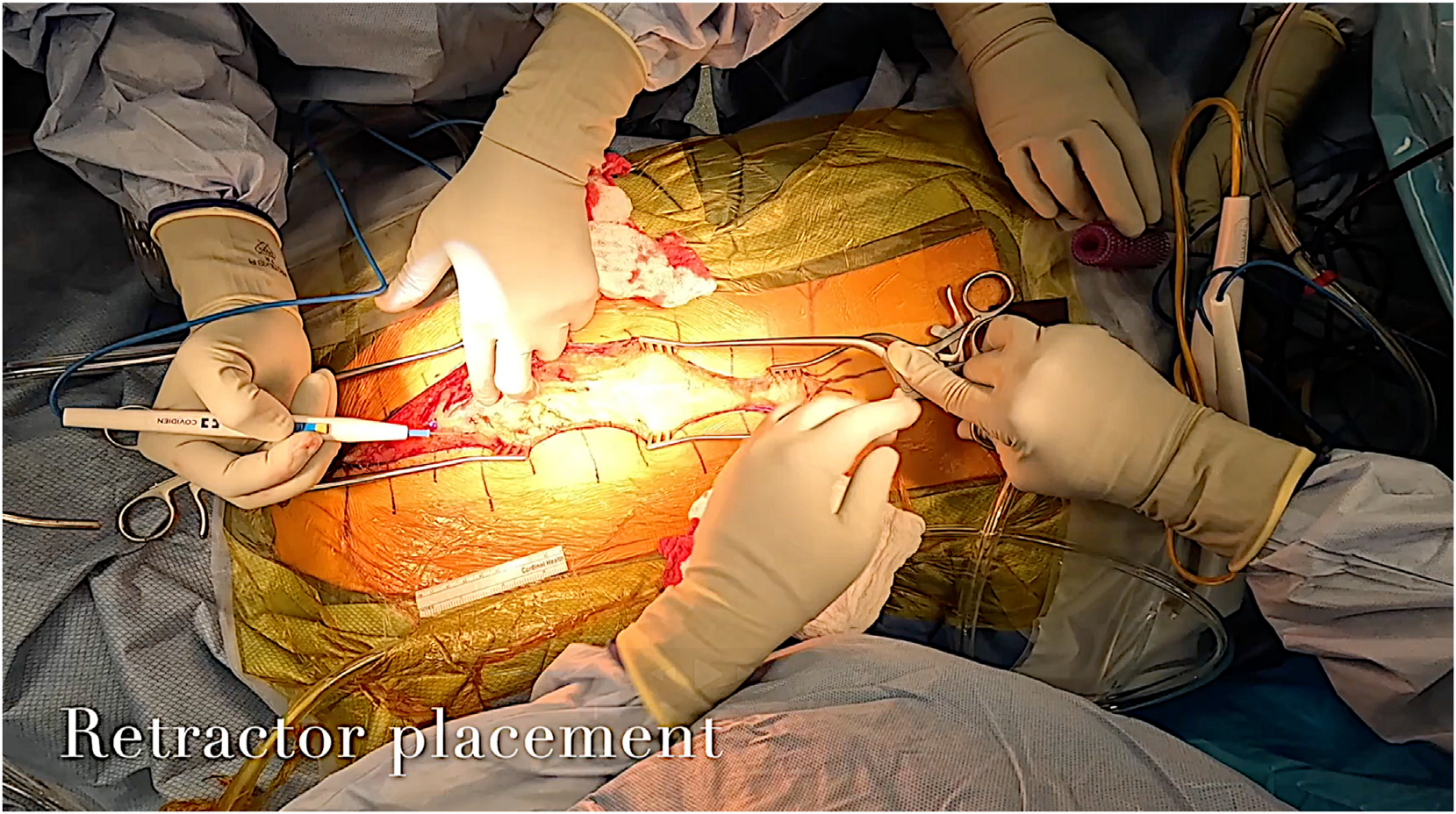
Ideal retractor sequence is demonstrated. A long-straight Weitlaner retractor is placed over an apically located short-straight Weitlaner retractor to distribute tension.

Manual digit-based retraction of the wound is utilized until enough soft tissue has been elevated to accommodate superficial retractors. Short straight Weitlaner retractors are then applied at the apices of the wound. These shorter retractors are positioned here to ensure their bases do not obscure the surgical field. Long straight Weitlaner retractors are then placed over the short straight retractors to reach the middle of the wound (Fig. 5). Superficial retraction with these self-retaining instruments aids hemostasis by compressing superficial dermal arteries. Dissection proceeds toward the spinous processes to the fascial layer. A Cobb elevator can be gently swept across the fascia to develop a continuous plane.

### Preservation of the supraspinous ligament

**Figure 6:**
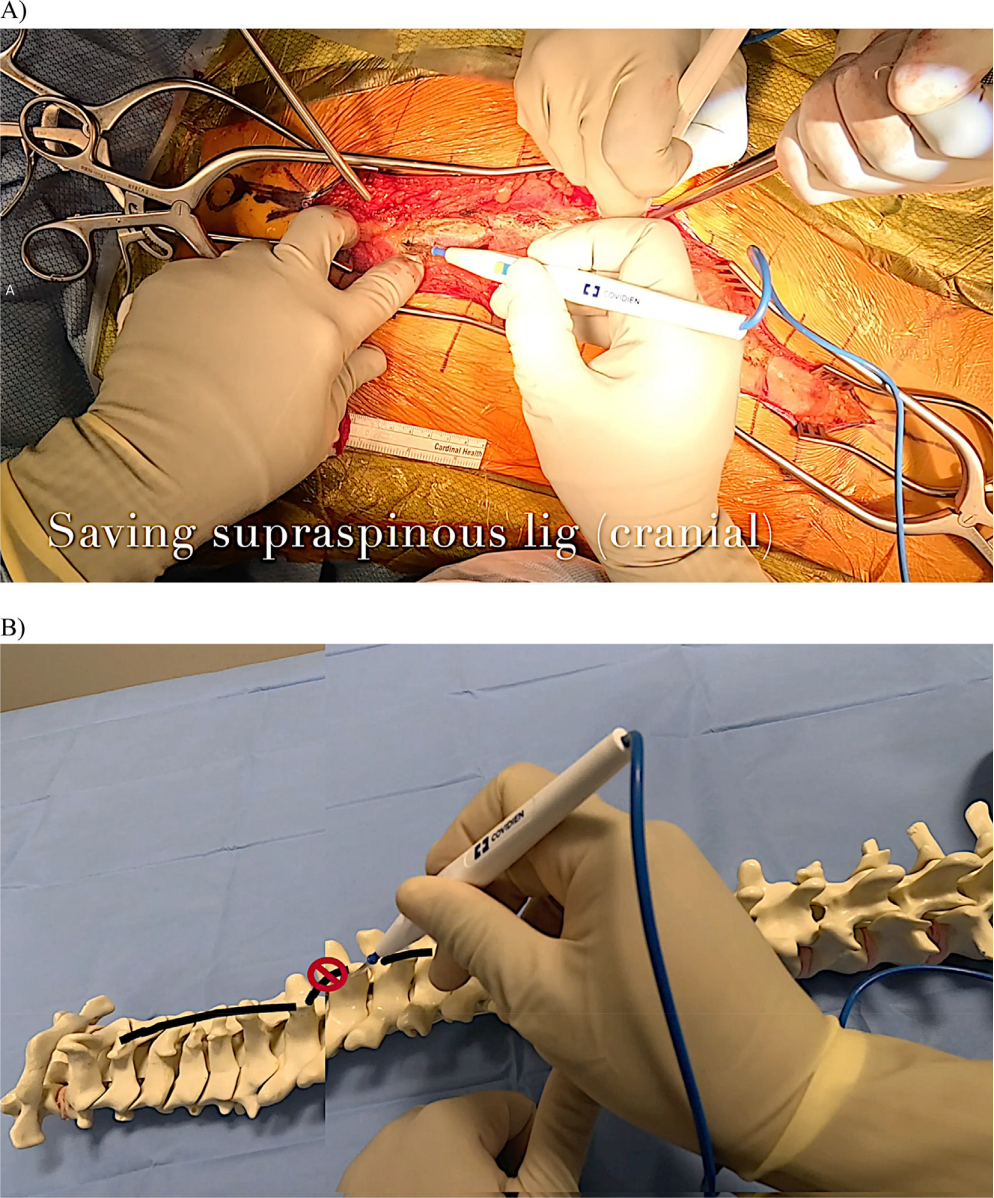
A) The cautery tip is dissecting at the “red-white” transition between the supraspinous ligament and the paraspinal muscles proximally near the UIV. B) Offset between vertebral levels is demonstrated, placing the supraspinous ligament at risk of transection or damage with errant dissection.

Dissection then proceeds to the “red-white” junction, which demarcates the transition between the supraspinous ligament centrally and the paraspinal muscles laterally (Fig. 6A). This is performed at the apex of the incision while preserving the supraspinous ligament, starting at least two or three levels lower than the upper instrumented vertebrae (UIV) cranially and at least beginning at the level of the lowest instrumented vertebrae (LIV) caudally. Preserving the supraspinous ligament cranially near the UIV is critical for mitigating proximal junctional kyphosis (PJK) [[18], [19], [20], [21]]. The offset among the spinous processes must be recognized to avoid unintentional transection of the supraspinous ligament (Fig. 6B).

**Figure 7:**
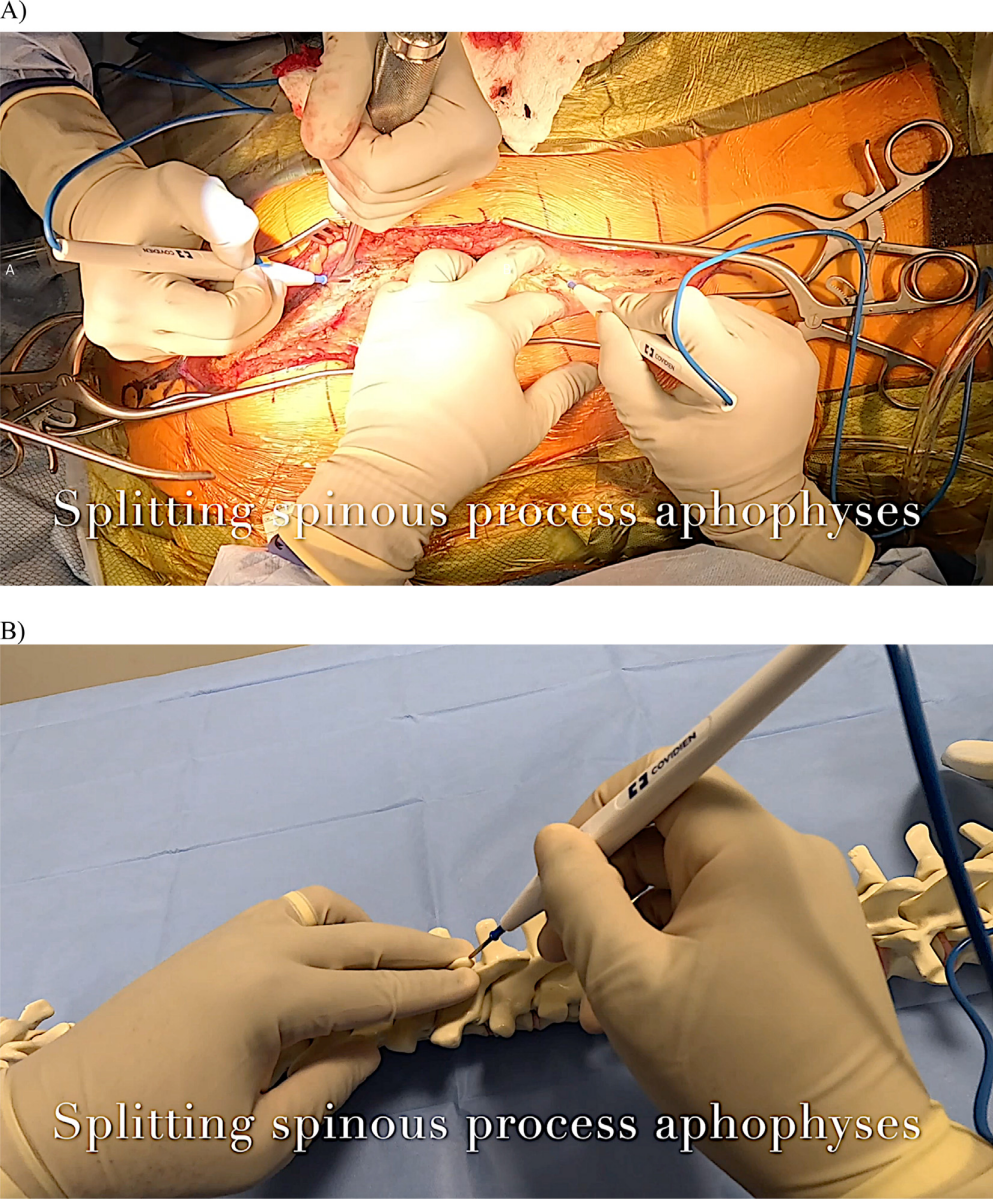
A) Manual finger pressure is applied bilaterally around the spinous process as cautery divides the supraspinous ligament and underlying apophysis. B) The same maneuver is demonstrated on a sawbones model.

Attention then turns to the spinous process apophyses. Cautery is used to divide the supraspinous ligament down to the tip of the spinous process, effectively splitting the apophyses. The split apophyses can then be mobilized laterally through blunt manual pressure or with cautery (Fig. 7A). One technique is to push on the apophyses with a finger covered by a laparotomy sponge, which may produce an audible ‘pop,’ indicating adequate mobilization. The apophyses are split sequentially, and dissection between them is performed with cautery, staying midline and riding on the tips of the spinous processes (Fig. 7B). This plane within the ligament is avascular. Significant bleeding encountered during this procedure may indicate that the paraspinal muscle has been violated.

### Subperiosteal dissection part I: spinous process, lamina, and pars interarticularis

There are various techniques for subperiosteal dissection. One method involves dividing the procedure into two parts: part I consists of a narrow dissection, while part II involves a wider dissection.

**Figure 8:**
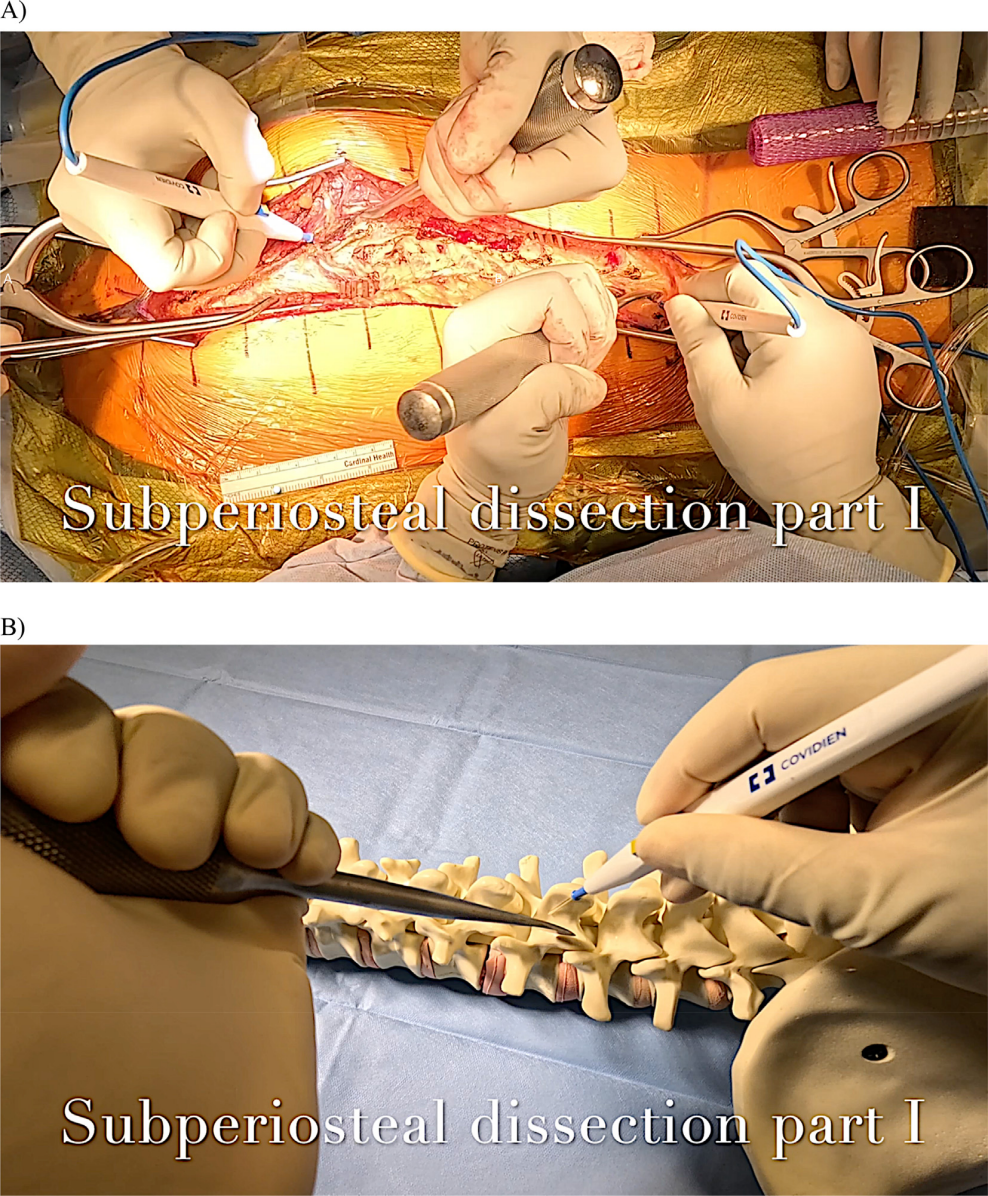
A) The Cobb elevator is gently positioned, aiming superiorly along the spinous process to facilitate subperiosteal dissection in the direction of the lamina. B) A sawbones rendition of the appropriate Cobb and cautery tip orientation to safely expose the lamina.

Both surgeons now utilize a Cobb elevator in their nondominant hand to aid in elevating the paraspinal musculature during subperiosteal dissection. Alternatively, a metal suction tip can substitute for a Cobb to facilitate soft tissue retraction. Suction tip retraction can assist the surgeon in achieving more rapid hemostasis if bleeding occurs during exposure. The thoracic spinous processes extend caudally in the sagittal plane. Sharp dissection initiates inferiorly at the tip of the spinous process and advances ventrally and superiorly toward the lamina (Fig. 8A). This contrasts with the dissection of the lumbar spine, where cautery should progress relatively horizontally from the spinous process outward toward the lamina. The surgeon must recognize that the interlaminar space is broader in the lumbar spine than in the thoracic spine, which can predispose one to potential durotomy.

Frequent repositioning of the Cobb's edge in the same orientation as the cautery tip is advised to maintain dissection within the subperiosteal plane. At this juncture, it is helpful to turn off the cautery and, once completely cooled, use the tip as a “sounder” to identify the orientation of the vertebrae's posterior elements if the surgeon is unsure of the osseous anatomy (Fig. 8B). The paraspinal muscles are elevated off the spinous process, lamina, and pars interarticularis. Dissection should not proceed lateral to the pars interarticularis at any level due to the high risk of injury to the exiting nerve root. In the upper lumbar spine, this can be particularly debilitating, as iatrogenic nerve root injury can cause significant lower extremity dysfunction, leading to a loss of mobility.

One should avoid laterally “scraping” the soft tissue sleeve with the bovie. Instead, a gentle touch should be employed with the cautery device to prevent ventral penetration of the neural elements. Errant movement of the cautery tip in the interlaminar space risks violating the underlying ligamentum flavum, which may lead to durotomies and potential spinal cord or spinal nerve root injuries.

**Figure 9:**
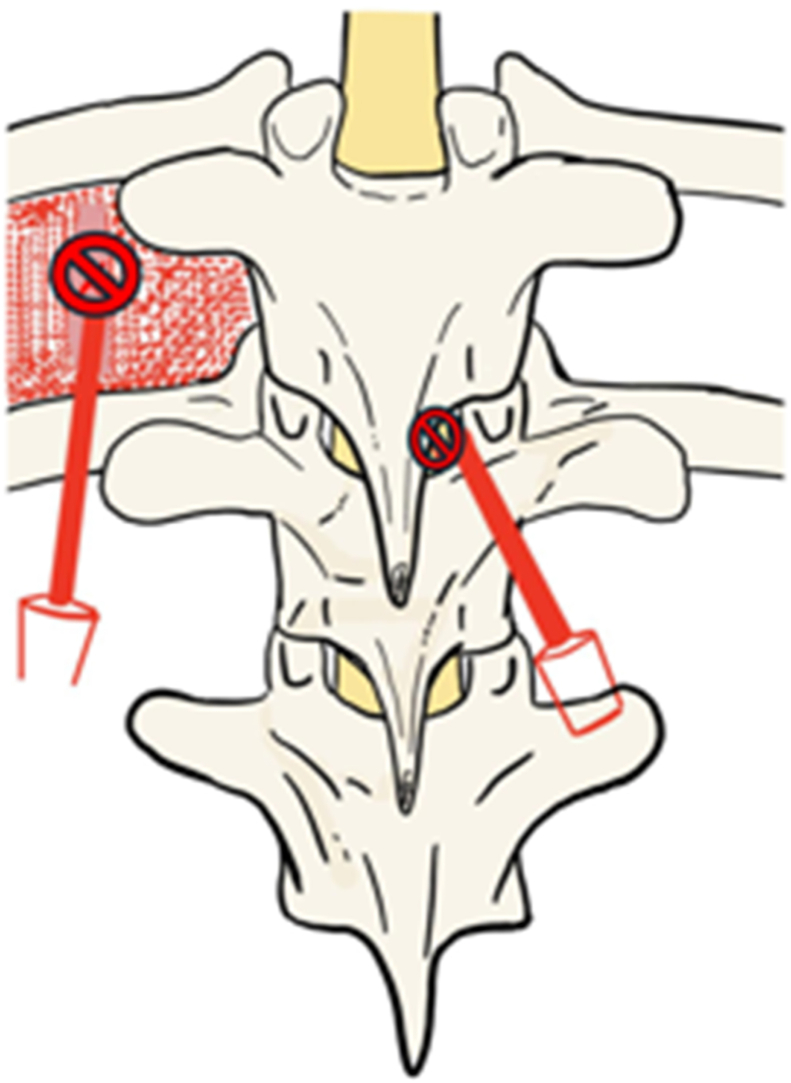
Errant electrocautery dissection ventral to the thoracic TP may lead to pneumothorax if dissection extends deep to the external intercostal muscles (dotted red lines) and pleura (dark red space). Another electrocautery tip medial to the medial border of the facet joints in the interlaminar space may lead to durotomy and spinal cord injury.

As more paraspinal musculature is elevated, lateral displacement of the soft-tissue sleeve becomes easier. Inadvertent cauterization of the muscle with the shaft of the electrocautery tip should be minimized to limit myonecrosis, which may contribute to postoperative paraspinal muscle atrophy and infection. Elevating the intervening muscle is critical for adequate visualization and eventual posterolateral osseous fusion. Complete dorsal visualization of the spinous process, the lamina, and the lateral border of the pars interarticularis should be obtained (Fig. 9).

### Subperiosteal dissection part II: transverse process and facet joints

**Figure 10:**
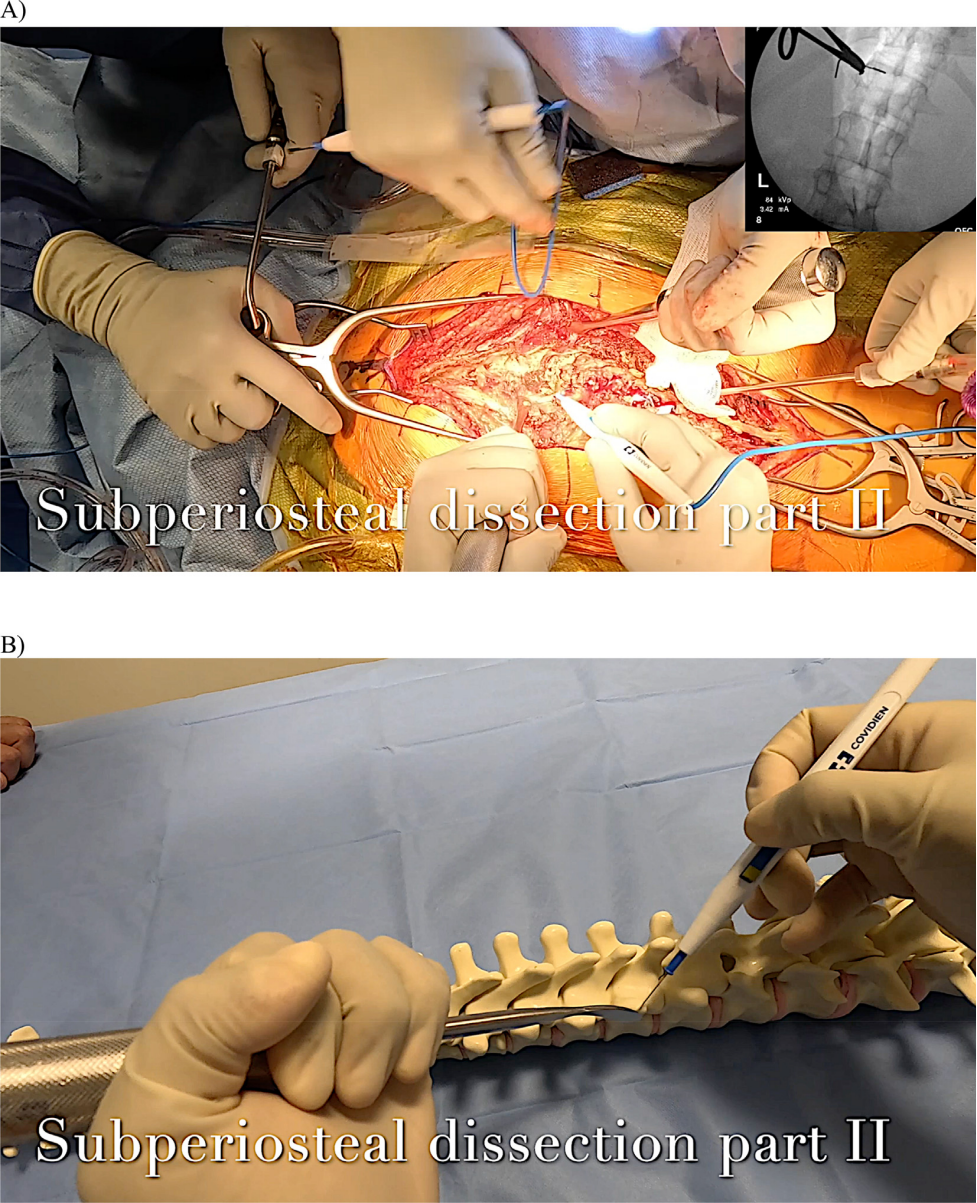
A) Demonstration of posterolateral dissection over the TP with the Cobb placed at the lateral tip of the TP in a patient B) Rendition of the same Cobb and cautery positions on a sawbones model.

Exposure of the facet joint and transverse process in the lumbar spine, as well as the transverse process in the thoracic spine, is then performed. The Cobb elevator can be positioned at the lateral aspect of the facet in the lumbar spine and the lateral aspect of the transverse process in the thoracic spine to provide lateral tension on the paraspinal muscles. Cautery is then used to extend the subperiosteal dissection from the lamina to the facet or transverse process, respectively (Fig. 10). In the lumbar spine, after exposing the facets, the Cobb laterally retracts the paraspinal muscles dorsal to the lumbar transverse process and subperiosteally exposes the transverse process. The tip of the cautery should not travel deep to the transverse process to avoid nerve root injury. The intertransversarii muscles are disconnected as multiple adjacent thoracic and lumbar transverse processes are exposed. The transverse processes should be exposed cranially, caudally, and laterally out to the tips. This is an appropriate time to attach a radiopaque marker to a vertebra to determine the UIV and LIV.

Unnecessary dissection ventral to the transverse processes (TP) carries the additional risk of pneumothorax in the thoracic spine. Air bubbles may be seen emanating from blood, or an audible ‘whoosh’ may be heard should this occur. There is an additional risk of violating the exiting nerve root and the accompanying vasculature by dissecting too deep to the distal edge of the TPs in the thoracic and lumbar spine. The changing morphology of thoracic TPs must also be recognized during dissection: TPs at T1 are wide and prominent laterally, whereas those at T11 and T12 are short and tuberculated. This contrasts with the relatively uniform morphology of the lumbar TPs. Additionally, lumbar transverse processes can be easily localized with a Cobb or by going just inferior and lateral to the adjacent facet joint. Dissection of the TP is complete when the superior, inferior, and lateral borders have been adequately outlined and freed of soft tissue.

**Figure 11:**
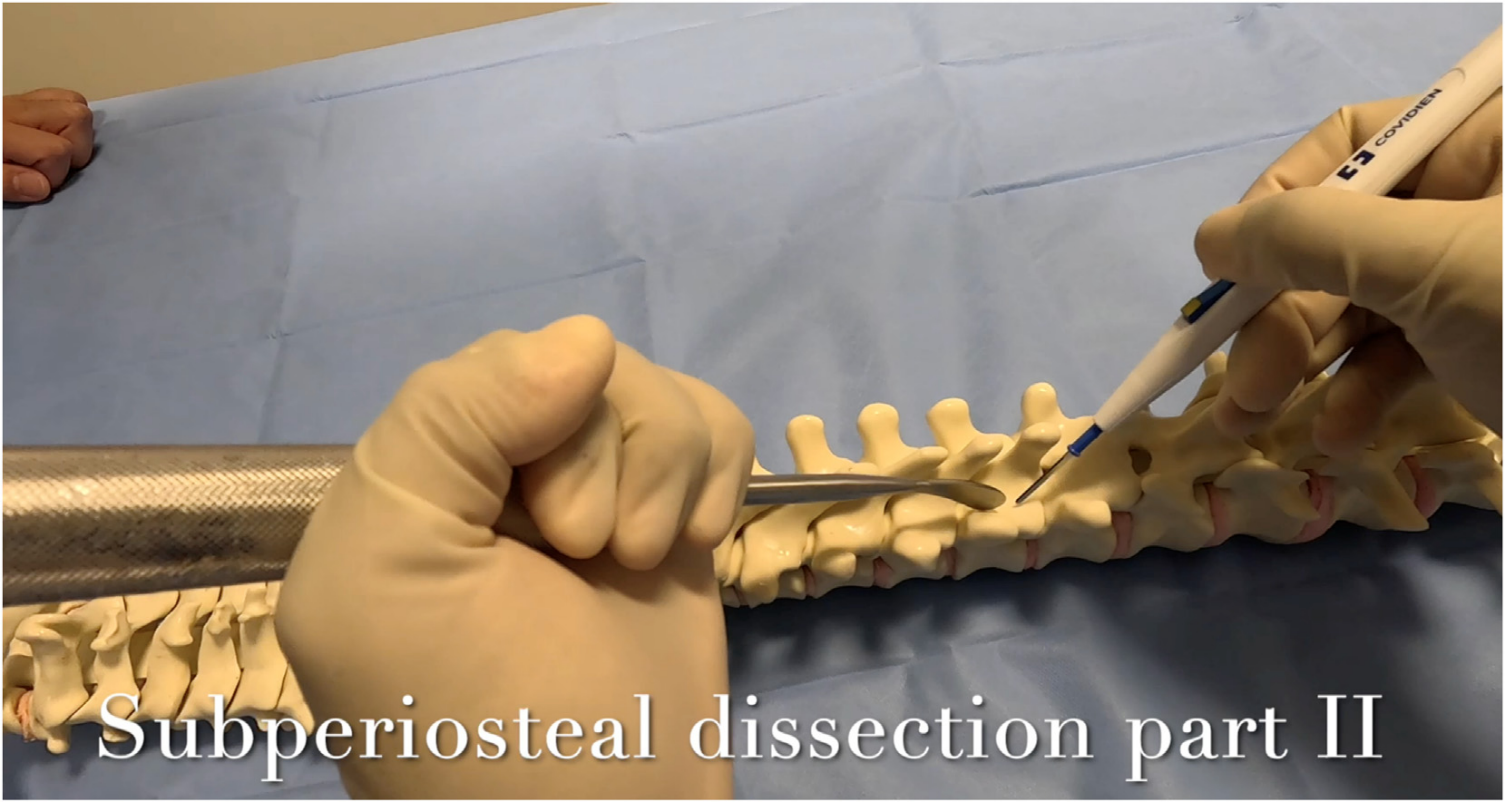
Sawbones demonstrate taking down the facet joint capsule beginning cranially.

Lastly, the facet joint capsules should be cleared to prepare for facetectomy. The appearance of the joint capsule, as well as the size and orientation of the thoracic and lumbar facet joints, varies by vertebral level. While the facet joints in the upper thoracic spine are small and thin, those in the lumbar spine are larger with thick, overlying “pearly white” capsular tissue. Exposure of the facet joints within the planned fusion construct requires the removal of all overlying soft tissue. The cautery tip works from cranial to caudal across the facet joint and never deviates medial to the facet joint (Fig. 11). Dissection medial to the medial aspect of the facet joint may violate the spinal canal. It is critical to identify the inferolateral corner and lateral border of the facet joint for eventual facetectomies and safe pedicle screw insertion.

The cranial facet at the UIV should be preserved, as should the caudal facet at the LIV. Violating the facet joint capsule around the UIV's SAP and the LIV's IAP may increase the risk of adjacent segment disease (ASD), potentially causing pain after PSF for IS [22]. Exposure of the facet joints is considered complete once the joints are free of overlying soft tissue that could obstruct eventual posterolateral fusion and once the lateral borders have been defined. Finally, a curette and lap sponge can be used to scrape away any charred material covering the posterior spinal column, after which the wound is thoroughly irrigated. The surgeons then prepare for facetectomies and pedicle screw or hook placement, ensuring clear anatomical landmarks are visible [23].

## Comparison to other methods

While specific steps may differ among pediatric spine surgeons, the posterior approach is performed without significant national variation. Furthermore, academic instruction for this approach has typically relied on self-study using established orthopaedic textbooks, such as Hoppenfeld's Surgical Exposures in Orthopedics: The Anatomical Approach. These written resources excellently cover the steps of dissection; however, surgical videos provide further nuance and detail, enhancing education. Recently, video-based resources accessed through websites like VuMedi or YouTube and compendiums such as the AAOS OVT and POSNAcademy have become more widely circulated among surgical trainees. These resources dramatically enrich case preparation through visualization of the surgical wound and relevant anatomy at various stages throughout the case.

This technique video and accompanying manuscript offer unique value because they provide a comprehensive review of the approach to prepare trainees for intraoperative success in an easily digestible format, along with an assessment tool for attending surgeons to identify critical preoperative knowledge deficiencies in residents (Appendix). Adequate preparation for these cases using this resource will hopefully limit blood loss, reduce operative time, and decrease the incidence of iatrogenic mistakes, potentially leading to improved surgical outcomes.

## Summary

This technique video highlights the principles of posterior thoracic spine exposure. An efficient, systematic subperiosteal dissection is crucial for intraoperative hemostasis. The supraspinous ligament should be preserved at the UIV+2/UIV+3 and the LIV. The subperiosteal dissection to reveal key osseous landmarks for safe pedicle screw placement may be completed in two phases. The first phase involves exposing the spinous process, lamina, and pars interarticularis. The second phase entails exposing the TPs and facet joints. Lastly, trainee surgeons should concentrate on preserving the adjacent facet joints to reduce the risk of junctional kyphosis and adjacent segment disease.

## Additional links

• **AAOS OVT**: Posterior spinal fusion, approach included, for idiopathic scoliosis
• **POSNAcademy:** Wound closure in non-idiopathic scoliosis
• **POSNAcademy:** Master's techniques posterior column osteotomies for pediatric spine deformity
• **POSNAcademy:** Two surgeon approach to posterior spinal fusion in the correction of neuromuscular scoliosis
• **POSNAcademy:** How and when to use hooks to improve deformity correction

## Author contributions

**Ravi R. Agrawal:** Writing – review & editing, Writing – original draft, Visualization, Validation, Supervision, Software, Project administration, Methodology, Investigation, Formal analysis, Conceptualization. **Keith Bridwell:** Writing – review & editing, Writing – original draft, Validation, Supervision, Resources, Methodology, Conceptualization. **Munish Gupta:** Writing – review & editing, Validation, Supervision, Conceptualization. **Blake K. Montgomery:** Writing – review & editing, Writing – original draft, Visualization, Validation, Supervision, Software, Resources, Project administration, Methodology, Investigation, Funding acquisition, Formal analysis, Data curation, Conceptualization.

## Consent for publication

A complete written informed consent was obtained from the patient, their guardian or legal representative for the publication of this study and accompanying images.

## Funding

Research reported in this publication was supported by the 10.13039/100006108National Center for Advancing Translational Sciences of the 10.13039/100000002National Institutes of Health under Award Number UL1 TR002345. The content is solely the authors' responsibility and does not necessarily represent the official views of the National Institutes of Health.

## Declaration of competing interests

The authors declare that they have no known competing financial interests or personal relationships that could have appeared to influence the work reported in this article.
