# Supplementary material for: Thoracic and Lumbar Spine Dissection for Pediatric Deformity
Source: J Pediatr Soc North Am. 2025 May 28;12:100213. doi: 10.1016/j.jposna.2025.100213 (PMC12317433; doi:10.1016/j.jposna.2025.100213)
Supplement: Multimedia component 4 [file mmc4.docx]

**Appendix.**

Questionnaire to be administered by faculty before and after a trainee uses this educational resource. Ideally, it should be provided prior to assisting with exposure of the posterior spinal column during PSF for IS. The correct answers are below.

1. Dissection of the thoracic spine should proceed in which of the following orders?
   1. Skin, fat, fascia, apophysis, spinous process, transverse process, pars interarticularis, facet joint, lamina
   2. Skin, fat, fascia, spinous process, apophysis, pars interarticularis, facet joint, lamina, transverse process
   3. Skin, fat, fascia, transverse process, facet joint, apophysis, spinous process, lamina, pars interarticularis
   4. Skin, fat, fascia, apophysis, spinous process, lamina, pars interarticularis, transverse process, facet joint
2. Unintentional dissection at the UIV interlaminar space risks what likely complication?
   1. Pneumothorax
   2. Nerve root injury
   3. Supraspinous ligament transection
   4. Spinal cord injury
   5. Adjacent segment degeneration
3. Unintentional dissection ventral to the thoracic transverse processes at T5 risks what likely complication?
   1. Pneumothorax
   2. Nerve root injury
   3. Supraspinous ligament transection
   4. Spinal cord injury
   5. Adjacent segment degeneration
4. Unintentional dissection lateral to the pars interarticularis at L1 risks what likely complication?
   1. Pneumothorax
   2. Nerve root injury
   3. Supraspinous ligament transection
   4. Spinal cord injury
   5. Adjacent segment degeneration
5. True or false: Significant bleeding is expected with dissection through the supraspinous and interspinous ligaments when exposing the spinal column.
   1. True
   2. False
6. True or false: The morphology of thoracic transverse processes are similar from T1 to T12.
   1. True
   2. False
7. Damage to the supraspinous ligament near the UIV is most likely to lead to what postoperative complication?
   1. Proximal junctional kyphosis
   2. Distal junctional kyphosis
   3. Adjacent segment disease
   4. Deep infection
8. What structures do NOT need to be exposed for safe placement of freehand pedicle screws at the UIV?
   1. IAP of UIV
   2. SAP of UIV
   3. SAP of LIV
   4. SAP of UIV+1
9. What vertebral structures must be completely dissected and visualized for safe freehand thoracic pedicle screw placement?
   1. Transverse process and the SAP
   2. Transverse process and the SAP
   3. Spinous process and the SAP
   4. Spinous process and the IAP
   5. Lamina and the SAP
10. The apex of the iliac crests typically correspond to what anatomic landmark?
    1. L1
    2. L2
    3. L3
    4. L4
    5. L5

**Answers**

1-d

2-c

3-a

4-b

5-b

6-b

7-a

8-b

9-a

10-d
